# Proteomic Analysis of Ferrochelatase Interactome in Erythroid and Non-Erythroid Cells

**DOI:** 10.3390/life13020577

**Published:** 2023-02-18

**Authors:** Chibuike David Obi, Harry A. Dailey, Yasaman Jami-Alahmadi, James A. Wohlschlegel, Amy E. Medlock

**Affiliations:** 1 Department of Biochemistry and Molecular Biology, University of Georgia, Athens, GA 30602, USA; 2 Department of Microbiology, University of Georgia, Athens, GA 30602, USA; 3 Department of Biological Chemistry, University of California Los Angeles, Los Angeles, CA 90095, USA; 4 Augusta University/University of Georgia Medical Partnership, Athens, GA 30606, USA

**Keywords:** erythroid, ferrochelatase, heme, heme biosynthesis, interactome, metabolon, mitochondria, non-erythroid, tricarboxylic acid cycle

## Abstract

Heme is an essential cofactor for multiple cellular processes in most organisms. In developing erythroid cells, the demand for heme synthesis is high, but is significantly lower in non-erythroid cells. While the biosynthesis of heme in metazoans is well understood, the tissue-specific regulation of the pathway is less explored. To better understand this, we analyzed the mitochondrial heme metabolon in erythroid and non-erythroid cell lines from the perspective of ferrochelatase (FECH), the terminal enzyme in the heme biosynthetic pathway. Affinity purification of FLAG-tagged-FECH, together with mass spectrometric analysis, was carried out to identify putative protein partners in human and murine cell lines. Proteins involved in the heme biosynthetic process and mitochondrial organization were identified as the core components of the FECH interactome. Interestingly, in non-erythroid cell lines, the FECH interactome is highly enriched with proteins associated with the tricarboxylic acid (TCA) cycle. Overall, our study shows that the mitochondrial heme metabolon in erythroid and non-erythroid cells has similarities and differences, and suggests new roles for the mitochondrial heme metabolon and heme in regulating metabolic flux and key cellular processes.

## 1. Introduction

Protein–protein interactions are an important post-translational regulatory strategy for controlling metabolic pathways. In some cases, protein–protein interactions modify activity and in others, facilitate channeling of metabolites to augment the flux towards specific biological processes [1,2,3,4,5,6]. This is the case for the heme biosynthetic pathway [7], where the first enzyme in the pathway 5-aminolevulinic acid synthase (ALAS) and the terminal enzyme ferrochelatase (FECH) interact with each other as well as other mitochondrial heme synthesis enzymes and additional proteins to regulate the pathway [8,9,10]. These proteins form the mitochondrial heme biosynthesis metabolon [11], thus facilitating the trafficking of substrates and products to prevent cellular damage due to the cytotoxic nature of intermediates and product as well as regulating the flux through the pathway [12].

Initially, the mitochondrial heme metabolon was identified in erythroid cell lines [9]. Developing erythroid cells have a very rapid and high demand for heme synthesis (~10^9^ molecules in mature erythrocyte) as they are specialized to make large quantities of hemoglobin and ultimately differentiate into mature erythroid cells [13]. In these cells, FECH, which catalyzes the insertion of iron into protoporphyrin IX [14], was shown to interact with different proteins for enzyme stability [15], as well as substrates and product transport [15,16]. With respect to iron delivery and homeostasis, FECH forms an oligomeric complex with mitoferrin-1 (MFRN1), a mitochondrial iron transporter, and ATP-binding cassette sub-family B member 10 (ABCB10) to channel iron imported into the mitochondria towards heme biosynthesis [9,15,16]. Additionally, FECH was found to interact with the erythroid-specific form of ALAS (ALAS2) and protoporphyrinogen oxidase (PPOX), the penultimate enzyme in the pathway. The interaction with ALAS2 likely regulates porphyrin precursor production, while the interaction with PPOX is for transfer of protoporphyrin IX to FECH [9]. Overall, these interactions are important for regulating flux through the pathway and protecting the cellular milieu from these potentially cytotoxic metabolites, which include porphyrins and iron.

Unlike erythroid cells, non-erythroid cells have a much lower demand for heme that is dependent on the changing hemoprotein needs of the cell. Thus, the regulation of heme synthesis in non-erythroid cells is different and more dynamic [17]. Investigation of the mitochondrial heme metabolon in non-erythroid cells has been much more limited. FECH has been shown to interact with progesterone receptor membrane component 1 and 2 (PGRMC1 and PGRMC2) in kidney, liver, and adipose cell lines and tissue. In these cells and tissue, PGRMC1 and PGRMC2 have been proposed to serve as a heme chaperone and sensor [10,18,19]. FECH interaction with PGRMC1 has been shown to decrease the enzyme’s activity in vitro [10] and thus may play a role in regulating heme biosynthesis via trafficking of the final product. Recently in *Saccharomyces cerevisiae*, interactions between FECH and the mitochondrial contact site and cristae organizing system (MICOS) suggest that proper localization of the mitochondrial heme metabolon is essential for heme synthesis and its regulation [20]. While these studies provide some insight, additional studies are required to dissect the subtle difference in the mitochondrial heme metabolon and its role in regulating heme synthesis in non-erythroid cells with very different heme demands.

To better understand the mitochondrial heme metabolon, we used affinity purification and mass spectrometry (MS) to study the interactome of FECH in different cell lines, including erythroid and non-erythroid model cell lines. We hypothesize that there is a set of core essential interactions in all cells. There are also unique interactions for erythroid and non-erythroid cells necessary to fulfill the unique requirements specific for the cellular needs based on development or environmental conditions. Our data highlight the variations of the mitochondrial heme metabolon in erythroid and non-erythroid cells and provide insight into factors that might contribute to how heme synthesis is regulated differently in these cells.

## 2. Materials and Methods

### 2.1. Cell Preparation, Affinity Purification, and MS Analysis

FLAG-tagged FECH construct was designed and transfected in three mammalian cell lines: murine erythroleukemia (MEL) (gift from Barry Paw), human embryonic kidney 293T (HEK) (ATCC No: CRL-1573), and human adrenocortical carcinoma (H295R) (ATCC No: CRL-2128) as previously reported [9]. Cells stably expressing FLAG-tagged FECH were validated by immunoblot analysis using anti-FLAG antibody (Sigma, St. Louis, MO, USA). MEL and HEK cells were cultured in DMEM with 25 mM glucose, 1 mM sodium pyruvate, and 4 mM glutamine (Cellgro, Manassas, VA, USA), supplemented with 10% FBS (Atlanta Biologicals, Flowery Branch, GA, USA) and 1% penicillin/streptomycin (Cellgro, Manassas, VA, USA). H295R cells were cultured in DMEM: F1 (50/50) (Cellgro, Manassas, VA, USA), supplemented with Nu-serum, ITS premix, and 1% antibiotic/antimycotic (Cellgro, Manassas, VA, USA). Cells stably expressing expression constructs were grown in media containing puromycin (5 µg/mL) to about 95% confluency (1.5 × 10^6^ cells in 10 175 cm^2^ flasks), harvested and lysed for mitochondria isolation. Isolated mitochondria were lysed for affinity purification of FLAG-tagged FECH as previously described [9].

Protein pellets were resuspended in 8M urea, 100 mM Tris pH 8.5 buffer, then reduced by the addition of Tris (2-carboxyethyl) phosphine (TCEP) to a final concentration of 5 mM by incubation for 30 min and alkylated by iodoacetamide to a final concentration of 10 mM for another 30 min at room temperature. Before protein digestion, the urea concentration was diluted to 2 M with 100 mM Tris. Then, LysC was added at 1:100 (LysC: protein) and digested for 4 h at 37 °C. Samples were further digested for 12 h at 37 °C with trypsin at 1:100 (trypsin: protein).

To stop the digestion, 5% formic acid was added to the samples. Next, peptides were desalted using C18 pipette tips (Thermo Scientific, Waltham, MA, USA) and reconstituted in 5% formic acid before being analyzed by LC-MS/MS. Tryptic peptide mixtures were loaded onto a 25 cm long, 75 μm inner diameter fused-silica capillary, packed in-house as described previously [21]. The gradient was delivered by a 140-min gradient using a Dionex Ultimate3000 nanoflow HPLC, separated at a flow rate of 200 nL/min, and eluted directly into a Thermo Orbitrap Fusion Lumos instrument. The data-dependent acquisition strategy consisted of a repeating cycle of one full MS spectrum (Resolution = 120,000), followed by MS/MS of the ten most intense precursor ions from the full MS scan (Resolution = 15,000). Raw Data analysis was performed using the IP2 suite of software tools (Integrated Proteomics Applications, San Diego, CA, USA). Database searching of the MS/MS spectra was performed using the ProLuCID (Prolucid Technologies, Ontario, Canada) algorithm (version 1.0) and a user-assembled database consisting of all protein entries from the human-specific protein sequence FASTA database. Peptide identifications were organized and filtered using the DTASelect [22] algorithm using a decoy database-estimated false discovery rate of <1%. Proteins were considered present in the analysis if they were identified by two or more peptides.

### 2.2. FECH-Prey Statistical Analysis

Contaminant Repository for Affinity Purification (CRAPome) [23] was used to analyze the enrichment of FECH interacting partners in our study cell lines (Table S1). This analysis was used to remove background contaminants from consistent interactors of FECH. The following number of biological replicates were analyzed for each cell line: MEL *n* = 2, HEK *n* = 4, and H295R *n* = 3. At the time of this analysis, the repository was limited to data for human, *S. cerevisiae*, and *Escherichia coli* proteins. Thus, FECH interacting partners identified from MEL cells, a murine cell line, were converted to human orthologs. The proteins’ UniProtIDs were first converted to gene names using SynGo [24] and UniProt [25]. The gene names were then converted to their corresponding human orthologs using Ensembl’s BioMart package in R [26]. Unmatched genes from this database were manually annotated. Then, enrichment of FECH interacting partners was computed using the CRAPome [23], as false positives were simultaneously eliminated. MEL, HEK, and H295R cells transfected with pEF1alpha FLAG biotag (empty vector) were used as negative controls, and for HEK cells, CRAPome controls were used as well. The enrichment profiles of FECH preys in different cell lines were represented as fold change (FC). The fold change value of these interacting partners was computed using CRAPome’s statistical models for scoring interactions, based on each protein’s sample and control spectral counts [23]. These CRAPome enrichment profiles produced high-confidence interactions which reflect the level of prey-bait interaction in different cell lines. UniProt gene names were used throughout instead of protein names or UniProt protein ID. Name of proteins discussed herein are listed in Table S2.

### 2.3. Protein and Pathway Enrichment Analysis of FECH Interacting Partners

For pathway enrichment and subsequent analysis of FECH interacting partners, a fold change threshold was placed to select only highly enriched proteins in each dataset. These threshold values were 2 for H295R and HEK cells, and 1.5 for MEL. Total proteins pulled down in MEL was much lower than in other two cell lines, thus, leading to the variation in their threshold values. JVenny was used to generate a comparative Venn diagram of proteins from all cell lines [27]. Subcellular localization of FECH interacting proteins was classified using a web portal, SubCellBarCode [28], MitoCarta 3.0 [29], and g:Profiler [30].

Calculated FC values of FECH prey from each cell line differ significantly with the highest values in MEL, HEK, and H295R being 21.5, 82.2, and 39.6, respectively. So, to effectively compare the enrichment of identified proteins from each cell line, their FC values were normalized using the formula stated below to produce a better bait-prey interaction comparison.

Normalized Protein X in Cell line A = log ((Protein X FC for Cell line A/ Highest Cell line A FC value) × 100)

Results obtained afterward are termed “normalized fold change” values. This normalization helped generate a comparable, cell line-dependent visualization of FECH-prey interactions. g:Profiler was used to analyze the pathway enrichment of identified FECH partners [30]. Biologic processes enriched are discussed and indicated herein in italics. DISPLAYR (Pyrmont, NSW, Australia) was used for data organization and plotting of heatmaps.

## 3. Results

### 3.1. FECH Interacting Partners Present in All Cell Lines

The mitochondrial heme metabolon was initially described in an erythroid cell line [9] where heme synthesis is rapid and heme demand is very high. However, heme biosynthesis is not exclusive to erythroid cells and in non-erythroid cells heme synthesis occurs at much lower levels in a more dynamic manner. Even with the differences in rate and overall heme levels, we hypothesized that there are FECH interactions, both direct and indirect, that are common to both erythroid and non-erythroid cells and that these compose the core FECH interactome in all cells. Thus, affinity purification and MS analysis was performed to identify proteins that interact with FLAG-tagged FECH in both erythroid and non-erythroid cells. We utilized H295R, a human adrenocortical carcinoma cell line, and HEK, a human embryonic kidney cell line, cells, as non-erythroid cell lines. Each of these cells requires heme for common (e.g., respiration) and unique (e.g., steroidogenesis) processes. We compared data from these non-erythroid cell lines with those of our previously collected erythroid, MEL, cell FLAG-tagged FECH data.

To identify proteins that are common contaminates in AP-MS experiments and calculate the fold change in our data sets, we utilized the Contaminant Repository for Affinity Purification (CRAPome) database and analysis tool [23]. To increase the stringency of analysis and further validate consistent interactions, we placed a threshold of the fold change of 2 for H295R and HEK cells, and 1.5 for MEL of enriched proteins in these datasets and also normalized the fold change for each cell line. Of note is that both non-erythroid cell lines have similar number of proteins identified compared to the erythroid cell line, which is a more specialized cell in terms of its function.

**Figure 1:**
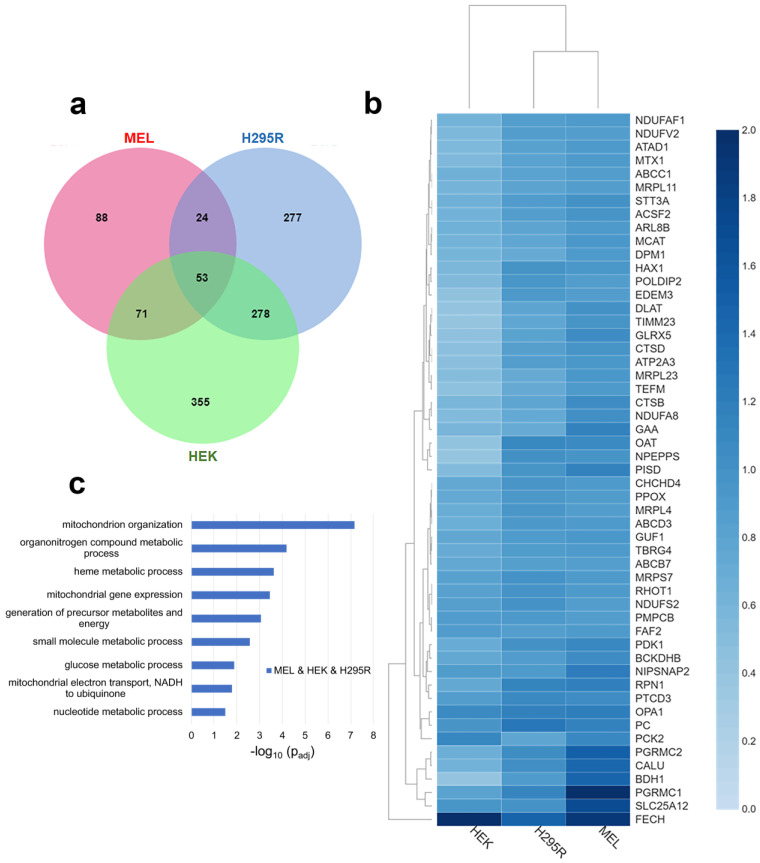
Comparative analysis of FECH interactome. (**a**) Venn diagram showing overlap of identified FECH interacting proteins across MEL (red), HEK (green), and H295R (blue). (**b**) Heatmap of the FECH interactome proteins common in all cell lines using their normalized fold change values. The color scale (2.0 to 0.0) represents their respective, normalized fold change values. Note: All values are greater than 0.0. Clustering of MEL and H295R is related to FECH expression in HEK cells. (**c**) Bar graph showing biological process enrichment profile of the 53 proteins found in the core FECH interactome in HEK, H295R, and MEL cells. *Y*-axis represents the biological processes while *X*-axis represents the −log_10_ of the adjusted *p*-values.

When comparing proteins that are above the stated threshold values in all three cell lines, 53 proteins, including FECH, were identified (Figure 1a). They represent approximately 7% of all enriched proteins in our dataset. Normalized fold change for these 53 proteins in each cell line is shown in the heatmap in Figure 1b. The subcellular localization of these proteins indicates that at least 74% are localized to the mitochondria (Table S3). While FECH is localized to the mitochondria, all proteins independent of their cellular localization were used for biological process enrichment analysis since mitochondria interact with other cellular organelles [31] and FECH has been recently reported to have alternate localizations [32]. Pathway enrichment profiles of the 53 proteins show that they are involved in multiple biological processes (indicated herein in italics), including *mitochondrial organization*, *organonitrogen compound metabolic process*, *heme metabolic processes*, *energy generation*, and *mitochondrial gene expression* (Figure 1c).

For *heme metabolic processes*, FECH, PPOX, ABCB7, ABCC1, PGRMC1, PGRMC2, and GLRX5 (Table S4) are proteins involved in or associated with heme metabolism found as part of the core FECH interactome. Most of these interactions have been identified before in the erythroid cell line [9] and these results emphasize their importance in non-erythroid cells. When comparing normalized relative fold change, PGRMC1 and PGRMC2 are highest in MEL cells, while PPOX and ABCB7 are similar in their normalized fold change in all cell lines.

Proteins involved in *mitochondrial organization* were also enriched in the core FECH interactome. These proteins include NIPSNAP2, OPA1, PISD, MTX1, RHOT1, CHCHD4, HAX1, NDUFA8, NDUFAF1, TIMM23, POLDIP2, NDUFS2, and PMPCB. Of these, OPA1 has been previously reported as a FECH protein partner in both mammalian cells [10] and *S. cerevisiae* [20]. Several of these proteins are involved in the mitochondrial contact site and cristae organizing system (MICOS) complex, which is a vital component of the mitochondrial intermembrane space bridging (MIB) complex [33].

With respect to *energy generation*, DLAT, GAA, NDUFA8, NDUFAF1, NDUFS2, NDUFV2, NIPSNAP2, and TEFM were all found as part of the core FECH interactome in all cell lines. Four of these proteins are part of the respiratory complex I (NADH: ubiquinone oxidoreductase), specifically NDUFS2, NDUFV2, NDUFA8, and NDUFAF1.

Other biological processes of note are *mitochondrial gene expression* and *metabolic processes*. For *mitochondrial gene expression*, MRPL11, MRPL23, MRPL4, MRPS7, PTCD3, TBRG4, and TEFM are found in the core interactome. Many of the *metabolic process* proteins overlap with those involved in *energy generation* but several of note are ABCD3, BDH1, BCKDHB, MCAT, OAT, PC, PCK2, PDK1, PISD, and SLC25A12 (Table S4).

### 3.2. FECH Interacting Partners Present in MEL and HEK or H295R Cells

**Figure 2:**
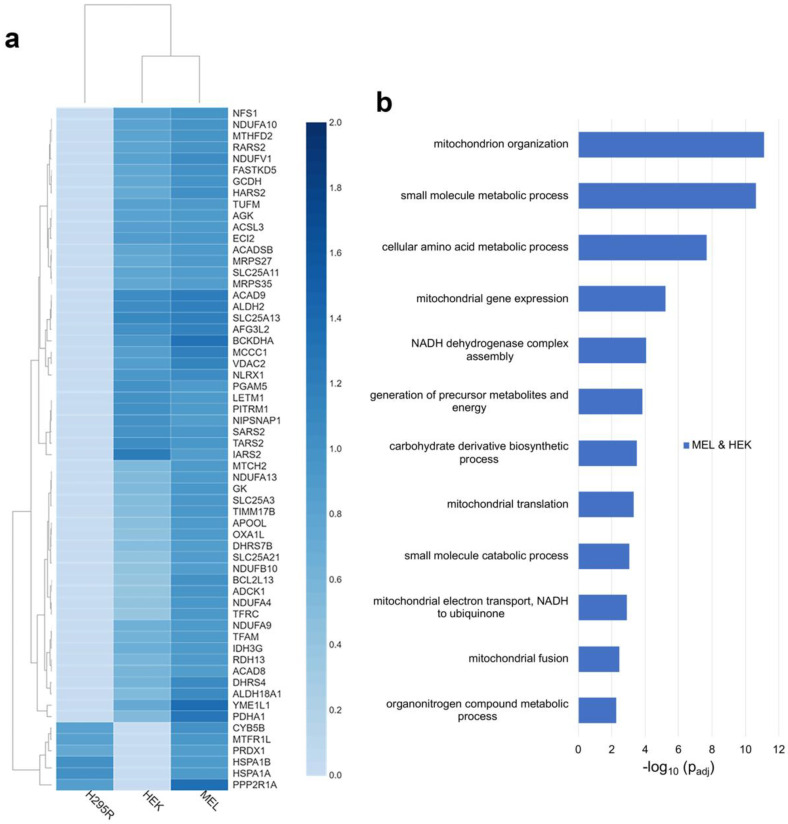
Analysis of distinct FECH interactomes in MEL and HEK or H295R. (**a**) Heatmap representing enrichment of mitochondrial proteins copurified with FLAG-tagged FECH in MEL and either HEK or H295R. (**b**) Biological process enrichment profile of proteins in the FECH interactome in MEL and HEK cell lines. *Y*-axis represents biological processes while *X*-axis represents the −log_10_ of the adjusted *p*-values.

To further define the FECH interactome, proteins identified as FECH interacting partners in MEL and HEK or H295R cells were analyzed. The FECH interactome partners found only in MEL and H295R cells were very limited, with only 24 proteins identified (Figure 1a). Of these, only 29% are localized to the mitochondria (Table S3). These included CYB5B, MTFR1L, PGS1, PRDX1, HSPA1A, HSPA1B, and PPP2R1A (Figure 2a). No biological process enrichment with strong links to mitochondria was noted. In addition to the mitochondrial proteins, there were several proteins identified with known links to heme, including GAPDH [34,35] and PCBP2 [36].

In MEL and HEK cells, a total of 71 proteins were found to interact with FECH only in these two cell lines (Figure 1a). Of these, 76% are localized to the mitochondria (Table S3) and their relative fold change for each cell line is shown in Figure 2a. Biologic processes enriched for these proteins were similar to those found in proteins from all three cell lines, though the enrichment differed (Figure 2b). For example additional proteins, including NDUFA10, NDUFA13, NDUFA4, NDUFA9, NDUFB10, and NDUFV1, involved in *complex I assembly and function*, were present in these two cell lines. For *mitochondrial organization*, additional proteins were found, including ADCK1, APOOL, YME1L1, and MTX2 which play essential roles in the maintenance of mitochondria crista junctions [33,37,38]. Biological processes unique to the proteins in MEL and HEK cells included *mitochondrial fusion*, *mitochondrial translation*, and *amino acid metabolic processes*. Proteins associated with *mitochondrial fusion* are ADCK1, AFG3L2, MTCH2, and TFRC, while those associated with *mitochondrial translation* are IARS2, MRPS27, MRPS35, RARS2, SARS2, TARS2, and TUFM. For *amino acid metabolism*, proteins included, ACAD8, ACADSB, ALDH18A1, BCKDHA, GCDH, HARS2, IARS2, MCCC1, RARS2, SARS2, SLC25A13, SLC25A21, and TARS2 (Table S4).

### 3.3. FECH Interacting Partners Unique to Non-Erythroid Cells

**Figure 3:**
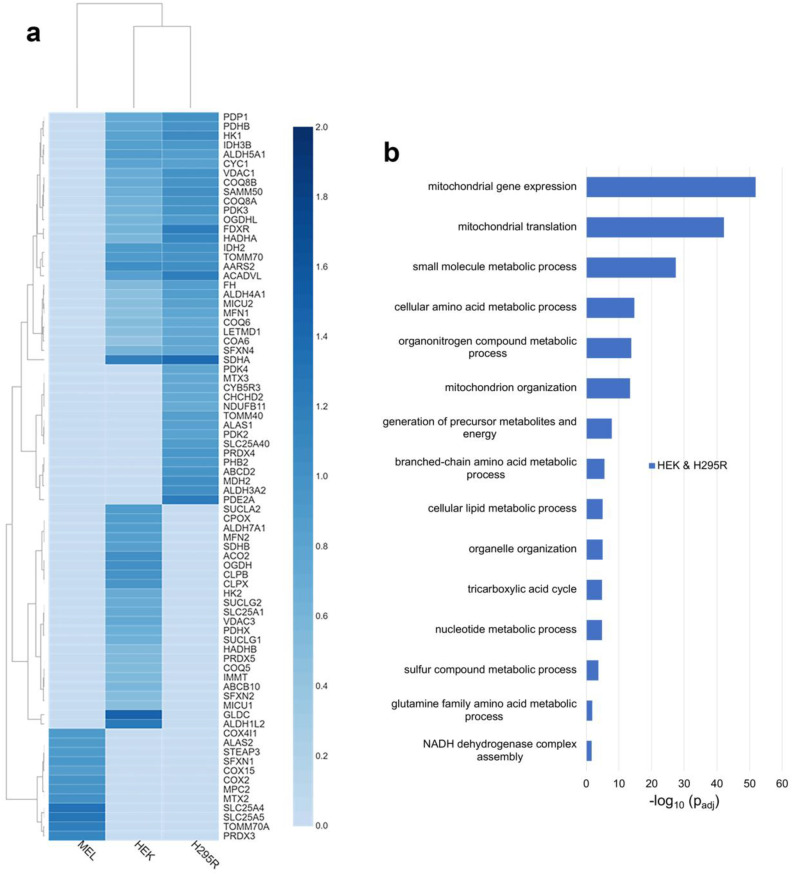
Analysis of distinct FECH interactomes in HEK and H295R, or unique to MEL, HEK, or H295R cells. (**a**) Heatmap representing select mitochondrial proteins found in both HEK and H295R cells, or unique to MEL, HEK, and H295R cell lines. The color scales (2.0 to 0.0) represent their respective, normalized fold change values. (**b**) Biological process enrichment analysis of proteins of the FECH interactome in non-erythroid cells, specifically HEK and H295R. *Y*-axis represents biological processes while *X*-axis represents the −log_10_ of the adjusted *p*-values.

To define the FECH interactome in non-erythroid cells, we compared the proteins found above the threshold in HEK and H295R cell lines. Specifically, 278 proteins were found to be in the FECH interactome in these two cell lines (Figure 1a), 64% of which were identified to be localized to the mitochondria (Table S3). Relative fold change of select mitochondrial proteins found in non-erythroid cells is shown in Figure 3a. Comparing the biologic process enrichment of the proteins in the FECH interactome in non-erythroid cells, many of the overarching processes found previously were still present and some more specific processes were found (Figure 3b). These include *cellular lipid metabolic processes*, *organelle organization*, *TCA cycle*, *sulfur compound metabolic processes*, and *glutamine amino acid metabolic processes*.

The *TCA cycle* enzymes found to be enriched in the non-erythroid cells are DHTKD1, FH, IDH2, IDH3B, NNT, OGDHL, PDHB, and SDHA (Table S4). With respect to *cellular lipid metabolism*, 37 proteins associated with this biological process were found to be part of the FECH interactome in non-erythroid cells, while 19 proteins associated with *sulfur compound metabolism* and eight proteins associated with *glutamine amino acid metabolism* were identified. Proteins associated with *organelle organization* were also enriched in non-erythroid cells, specifically 82 proteins in total. This is of note due to the proposed route of heme trafficking via organelle interactions [19] in some non-erythroid cells. A complete list of proteins found in non-erythroid cells and biological process enrichment can be found in Tables S3 and S4.

### 3.4. Cell Specific Interacting Partners of FECH

Since genes are expressed differentially in cells depending on their functions and environments, certain proteins of FECH interactome are likely to be exclusive to specific types of cells. Several of the unique proteins to MEL cells are ALAS2, COX15, and STEAP3 (Figure 3a). ALAS2 is expressed only in erythroid cells and has previously been shown to interact with FECH [9]. COX15 is involved in the synthesis of heme a from heme b [39,40], while STEAP3 is iron transporter and its deficiency is associated with hypochromic microcytic anemia [41]. Select other mitochondrial proteins only in the MEL cell line are shown in Figure 3a with a complete list in Table S3.

Unique to H295R cells, several proteins are of note. ALAS1, the form of ALAS that catalyzes the first step of heme biosynthesis in all cells other than developing erythroid cells, was found to be in the FECH interactome. Two additional proteins related to cytochrome P450-mediated processes were noted, specifically CYB5R3 and CYP21A2 (Figure 3a and Table S3).

In HEK cells, SUCLA2, ABCB10, VDAC3, OGDH, CPOX, IMMT, PDHX, MFN2, MCU, CLPX, CLPB, and GLDC are some of the mitochondrial proteins identified in the FECH interactome (Figure 3a). Of these, MFRN2, ACO2, SDHB, SUCLA2, CPOX, CLPX, and CLPB are moderately enriched with a relative fold change value of 1, unlike GLDC and ALDH1L2 with higher fold change values of 1.5 and 1.3, respectively. Some of these proteins are directly or indirectly involved in heme metabolism. SUCLA2, which is involved in the synthesis of succinyl-CoA, has been previously reported to interact with ALAS2 and FECH [9,42,43]. CPOX, the antepenultimate enzyme in the heme biosynthetic pathway, has been proposed to be part of the heme metabolon but its interaction in erythroid cells was unclear [9]. MFRN2 is a mitochondrial iron transporter [44] and its homolog MFRN1 has been shown to interact with FECH [16]. CLPX, a mitochondrial AAA+ unfoldase, has been demonstrated in previous studies to regulate the heme biosynthetic pathway [45,46]. IMMT, a key component of MICOS, was shown to interact with FECH and play a role in regulating porphyrin homeostasis [10,20]. The presence of GLDC in the FECH interactome is novel and interesting, with it having the highest relative fold change. A non-mitochondrial protein found unique to HEK cells is HMOX2, the constitutive heme degradation enzyme in mammalian cells.

## 4. Discussion

Both the cellular demand and the rate of synthesis of heme vary considerably depending on the cell type. Developing erythroid cells epitomize this since individually they need to synthesize over 10^9^ molecules of heme in a short duration [47]. Other cells such as hepatocytes and cells of steroidogenic tissue must respond to metabolites and stimuli to make heme for cytochromes P450-mediated processes when needed and degrade hemoproteins when demand diminishes [48]. Most other cells require less heme for energy production via cellular respiration and other necessary heme mediated processes. However, the importance of heme synthesis even at low levels is clearly demonstrated in some neurodegenerative diseases [49]. The regulation of heme synthesis to fulfill the differing cellular needs is not entirely understood and likely dependent on transcriptional, translational, and post translational processes. One post translational mechanism that regulates heme synthesis and homeostasis is protein–protein interactions of the mitochondrial heme metabolon. This metabolon has been investigated primarily in developing erythroid cell lines and some of the components have been identified and studied. However, since the developing erythron is uniquely adapted to produce heme for hemoglobin and not diverse cellular processes, the variety of proteins and their abundance in these cells have limited identification of the global or essential proteins within the mitochondrial heme metabolon. Thus, characterizing the metabolon from diverse cells will begin to establish the basic, necessary, or core, components in different cell types.

Herein, we have presented data from multiple affinity purification and MS experiments and analyses to determine the interactome of FECH, a core component of the mitochondrial heme metabolon. We have identified a large number of proteins in the FECH interactome, either overlapping in multiple cell lines or unique to each cell line. Interestingly, not all protein partners identified were mitochondrially localized proteins. These interactions may arise for inter-organelle interactions or proteins with multiple cellular localizations. The FECH interactome consists of both direct and indirect interactions and data herein do not differentiate between these interactions. The FECH interactome instead is a multiprotein complex of which FECH is a component that likely interacts with other multiprotein complexes, hence, the high number of interacting proteins. It should be noted that our current data do not rule out the possible existence of multiple, unique FECH interactome complexes. Additional reciprocal studies will be necessary to further define these interactions and multiprotein complexes partners.

Key functions of the mitochondrial heme metabolon are in substrate channeling, product delivery to proteins or cellular compartments, and regulation of cellular heme homeostasis. With respect to substrate channeling, the interaction between FECH and PPOX was previously proposed [50,51] and more recently found to exist in a developing erythroid cell model [9]. Herein, we have shown that PPOX is a core component of the FECH interactome in all cell lines investigated. The interaction between FECH and PPOX is likely direct and necessary to protect cellular components from the reactive metabolite protoporphyrin IX. Interactions with proteins involved in cellular iron transport and homeostasis, including ABCB10, MFRN1, MFRN2, ABCB7, and GLRX5, have previously been shown [9,15,16] and were found in our datasets. The fact that some of these proteins such as ABCB10 are only found in HEK cells in our study, while previously identified in developing erythroid cells, is due to the fold change cutoff used in our study necessary to filter the large dataset. Several other proteins involved in iron metabolism, including STEAP3, HSPA9 and FTL, were identified as components of FECH interactome in different cell lines (Figure 3a and Table S3). HSPA9, along with ABCB7 and GLRX5, play crucial roles in the metabolism of the iron-sulfur cluster [52,53,54], a cofactor of human FECH [55]. The function of these proteins in the interactome and the connection to human diseases, including sideroblastic anemia [56,57], are areas for further studies.

Other heme biosynthetic enzymes, including CPOX, ALAS1 and ALAS2, were also found to be components of the FECH interactome. Previous work to determine if CPOX, PPOX, and FECH interact were inconclusive [9]. Data from HEK cells herein suggest that CPOX is a component of the FECH interactome and likely is necessary for channeling substrate to PPOX. The presence of ALAS enzymes, both ALAS2 and ALAS1, in different cell lines in the FECH interactome suggests that an interaction between the first and last step of the pathway is a consistent interaction. The presence of ALAS2 in the mitochondrial heme metabolon was previously noted [9] and confirmed in developing erythroid cell lines herein. The presence of ALAS1 in the FECH interactome in H295R cells was a novel finding and suggests that the interaction between FECH and ALAS proteins is not unique to developing erythrons, but is conserved and important for regulating flux through the pathway in all cell types. Although not reported herein, ALAS1 was found in the HEK dataset, but did not meet the fold change threshold set for these studies. Related are proteins involved in regulating the function of ALAS1, such as CLPX and LONP1 [45,46,58,59] which were also found to be components of FECH interactome in HEK cells and the two non-erythroid cell lines, respectively (Figure 3a and Table S3).

The metabolites succinyl-CoA and glycine are substrates for the ALAS enzymes and their origins for heme synthesis have been recently investigated and differ depending on the cellular demand for heme. In developing erythroid cells, glutamine converted to glutamate and then to succinyl-CoA and extracellular glycine are the sources of substrates [8,60]. This is necessary as otherwise heme production would deplete cellular levels of glycine and succinyl-CoA. Alternatively, it has been shown that succinyl-CoA from the TCA cycle can fulfill the demand in non-erythroid cells [8,61]. We found multiple TCA cycle enzymes as part of the FECH interactome, particularly in non-erythroid cells, suggesting that the TCA cycle metabolon [4] may interact with the mitochondrial heme metabolon to coordinate flux through both pathways. These interactions are supported by recent work that demonstrated heme synthesis and homeostasis modulates the TCA cycle and oxidative metabolism [62]. Another interesting, and unexpected, finding was that one protein found in high abundance in HEK cells is GLDC which catalyzes the decarboxylation of glycine [63]. GLDC is expressed in certain non-erythroid tissues such as the brain, kidney, and liver [64]. Since GLDC degrades glycine and is part of the FECH interactome, its role in heme metabolism is worth exploring.

The biological process *mitochondrial organization* was consistent throughout our data in all cell lines for proteins of the FECH interactome. Previous work in both mammalian cells and *S. cerevisiae* identified interactions between proteins of the MICOS and FECH and showed that they were necessary for heme homeostasis [10,20]. Interestingly, *organelle organization* was a biologic process enriched in the FECH interactome in non-erythroid cells. Mitochondrial interactions with the ER have been proposed to play a role in heme trafficking from the mitochondria to the nucleus [19,65]. The further interactions between proteins of the MICOS and MIB as well as mitochondrial dynamics and mitochondrial associated membranes detailed herein further connect heme production to mitochondrial structure, function, dynamics, and interaction with other organelles.

With respect to trafficking of heme, the specific protein or proteins involved in this process are still unclear. Two proteins that have been proposed to act as heme chaperones are PGRMC1 and PGRMC2 [10,19,66,67]. Both are hemoproteins with reported localization to the mitochondrion and ER. PGRMC1 has been previously identified to interact with FECH and regulate its activity in vitro. Additionally, it has been shown to have the capacity to transfer heme to an apo-hemoprotein, making it a plausible heme chaperone [10]. Recent work with PGRMC2 shows that knockout of PGRMC2 in adipose tissue causes defective nuclear heme trafficking [19]. In this report, PGRMC1 and PGRMC2 were identified as part of the core FECH interactome found in all cells. This observation provides support for a model where PGRMC1 and PGRMC2 play roles in heme trafficking and/or sensing outside of the mitochondria. It is of note that PGRMC1 and PGRMC2 in erythroid cells was shown to interact with MICOS [10], thus linking heme export from mitochondria to mitochondrial organization via MICOS. Interestingly, we also found proteins involved in the electron transport chain, either core proteins or assembly factors enriched in the FECH interactome. It seems likely that heme destined for mitochondrial hemoproteins may utilize alternate proteins for trafficking and that the mitochondrial heme metabolon may not need to be associated with MICOS. Further studies on these proteins and their role in heme trafficking are needed.

In terms of the electron transport chain proteins found in the FECH interactome, those of complex 1 were surprising considering complex 1 does not have a heme cofactor. Several possibilities as to the cellular role of these interactions exist. First, during the reaction catalyzed by FECH, two protons are released from protoporphyrin IX upon iron insertion. Given the large numbers of heme molecules produced during erythropoiesis in developing erythroid cells, this could significantly contribute to the mitochondrial membrane potential. Interactions between FECH and complex 1 could occur in such a manner to translocate these protons to the inner membrane space to be utilized for ATP synthesis. An alternative possibility is that this interaction is a mechanism to coordinate electron transport chain production and assembly with heme production. For the production of the electron transport chain, transcription and translation of both nuclear and mitochondrially encoded proteins, as well as assembly, delivery, and insertion of multiple cofactors, require a high level of coordination between different cellular compartments. For such processes, the metabolon and/or heme may be key factors. Interestingly, enrichment of proteins involved in the biologic processes *mitochondrial gene expression* and *mitochondrial translation* were found in our data. It is unclear what role heme synthesis or hemostasis may play in mitochondrial gene expression and translation though a role in electron transport chain production and assembly is a possibility, thus worthy of investigation.

**Figure 4:**
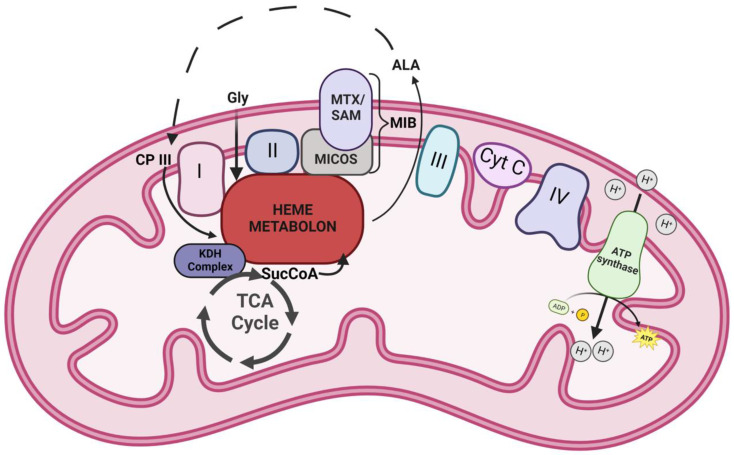
Model of FECH interactome. A schematic representation of the heme metabolon and its associated complexes in the mitochondria. Icons labeled I-IV represent the complexes of electron transport chain. Other abbreviations include Cyt C—cytochrome c, Gly—glycine, ALA—5-aminolevulinic acid, CP III—coproporphyrinogen III, SucCoA—succinyl-CoA, KDH—α-ketoglutarate dehydrogenase, MTX—metaxin, SAM—sorting and assembly machinery component, and MICOS—mitochondrial contact site and cristae organizing system. Dotted lines represent stages of the heme biosynthetic pathway that occur outside the mitochondria.

In conclusion, data presented herein provide additional insight on the composition and role of the mitochondrial heme metabolon in different cells from the perspective of the FECH interactome (Figure 4). Our findings reinforce previous interaction studies and also open new areas to investigate. New areas include the connections between mitochondrial organization, dynamics, and interactions with other organelles, as well as the TCA cycle metabolon, to cellular heme homeostasis, and role of the labile heme in regulating these diverse biologic processes.

## Data Availability

Data supporting the reported results can be found in Supplemental Tables S1–S4. The protein interactions from this publication have been submitted to the IMEx (http://www.imexconsortium.org (accessed on 17 February 2023)) consortium through IntAct [68] and assigned the identifier IM-29649.

## Supplementary Materials

The following supporting information can be downloaded at: https://www.mdpi.com/article/10.3390/life13020577/s1, Table S1: Raw Data; Table S2: Genes and Protein Names; Table S3: Crapome Filtered Data; Table S4: Biological Process Enrichment.
